# A Cultural and Natural History of the Fly

**DOI:** 10.1371/journal.pbio.0050135

**Published:** 2007-05-15

**Authors:** Andreas Keller

*Theater of Insects*, an entomology book from 1658, refers to flies as “little creatures so hateful to all men.” Most people's attitude towards dipterans has not changed much since, but maybe Steven Connor's excellent new book about the role of flies in culture and myth will help transform the fly's reputation.

**Figure:**
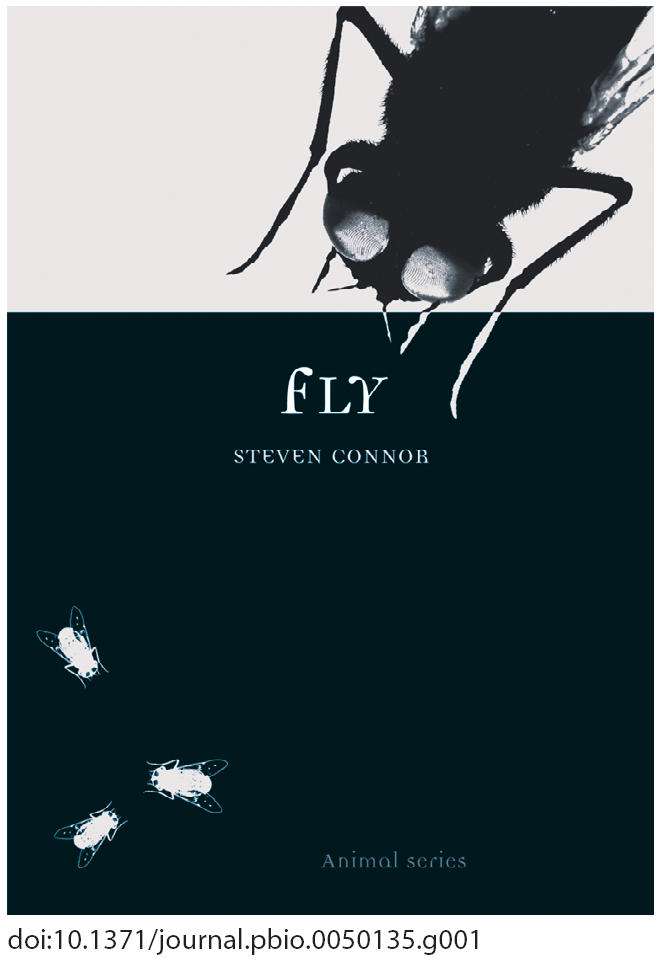
Connor S (2006) Fly. Chicago: Reaktion Books. 224 p. ISBN 1861892942. US$19.95.

In *Fly*, Connor tells us that the fly is loathed universally because it “takes its pleasure promiscuously, restlessly, unswervably, unashamedly.” Flies trample on their food and are so single-minded when it comes to reproducing that Aristotle remarked on the difficulty of pulling copulating flies apart. This carefree lifestyle offended human sensibilities. Flies became subjects for moral allegories about the consequences of pleasure-driven lives that often end with flies singed in flames or drowned in wine. Flies seem especially irresponsible when compared with social insects: ants and bees collect food and store it for the future or to feed their young and—at least the infertile workers—never copulate.

Because of their frivolous life, flies were used to signify sin throughout history. The capacity to command flies is the mark of the devil. Satan's lieutenant Beelzebub—the lord of the flies—is portrayed in a drawing from 1863 (reproduced in *Fly*) as a fierce fly-like creature with the skull and crossbones symbol on its wings. Satan himself, as well as many alleged witches and even Loki, the Nordic god of mischief, all change occasionally into flies. In their fly form, they have access to houses where they steal, torment, and seduce. The situation became even worse for flies when their role in transmitting diseases was discovered in the 19th century. A ruthless campaign for their extermination was started and books like *The House Fly: A Slayer of Men* and *The Reduction of Domestic Flies* urged readers to consider killing flies a moral duty.

Yet for all the aspersions cast upon the fly, the very qualities that inspired derision also stirred affection and respect. For the same reasons for which they were demonized, flies were sometimes poetically elevated to a symbol of liberty because “Each fly is king of his own country. He knows no laws or conventions…He has no work to do—no tyrannical instinct to obey… what freedom is like his?”

For many scientists, the word “fly” refers to a single species, Drosophila melanogaster. For Connor, “fly” refers to all members of the order Diptera, and the book also includes references to insects that are flies only by name, like butterflies or mayflies. Like others, Connor is confused by the unfortunate situation that the term “fruit fly” refers to several species. Therefore, the beautiful image illustrating the discussion of the fruit fly's use in genetic research on page 153 depicts a fly not well known to geneticists. It is the research on Drosophila melanogaster and other flies that, in Connor's narrative, exonerates the fly. The development of the microscope and other scientific advances showed the perfection, variety, and beauty of native and exotic flies. The intricate design of the fly's compound eye and the fly's elegant maneuvering during flight fascinated many early naturalists. The increased knowledge of flies and their natural history put an end to beliefs that demeaned flies, such as the idea that they could be generated spontaneously from mud or that they may not be created by the same God as higher animals and humans. Research has also explained some of the behaviors for which flies had been despised. It was found that flies have sugar sensors in their feet and thus “the fly's habit of trampling across its food is purposive and investigative rather than slovenly.” The biggest change in the public perception of flies came through genetic and developmental research in Drosophila melanogaster. In a century of research, many basic biological principles were discovered in Drosophila melanogaster, and it is now probably better understood than any other organism. Through the massive research effort directed at it, the lowly fly has become a “representative of all living forms.”

*Fly* identifies interesting connections between historical texts and modern fly research. Aristotle's intuition that fly offspring are “never identical in shape with the parents, but a something imperfect” was confirmed in the early 20th century by the first fly geneticist, Thomas Hunt Morgan, who found many spontaneous anatomical variants in his fly stocks at Columbia University in New York. Connor also remarks on the irony that flies have traditionally served as a symbol of brevity of mortal existence, yet recent studies, like the discovery of the methuselah gene, put flies in the center of longevity research. There are other areas in which modern science can comment on historical texts about flies, and each fly researcher could probably add one example to this book. Those that study learning and memory in flies have shown that Pliny the Elder was wrong in his opinion that no creature is “less teachable or less intelligent” than the fly. Those that study the courtship songs that Drosophila produce by vibrating their wings may not agree that the sounds they make seem “like the opposite of meaningful speech” and that flies ”have no voice and no language” (Aristotle).

It is not the objective of *Fly* to discuss Drosophila research. Instead it gives a fascinating tour through the role of flies of all species in culture and myth. Even if until now, your main interest in flies was how their embryos develop, you will enjoy reading this book—be it only to find out how many blowfly larvae it takes to devour the carcass of a horse as quickly as a lion.

